# Inhibition of PINK1/Parkin-dependent mitophagy sensitizes multidrug-resistant cancer cells to B5G1, a new betulinic acid analog

**DOI:** 10.1038/s41419-019-1470-z

**Published:** 2019-03-08

**Authors:** Nan Yao, Chenran Wang, Nan Hu, Yingjie Li, Mingqun Liu, Yuhe Lei, Minfeng Chen, Liping Chen, Chen Chen, Ping Lan, Weimin Chen, Zhesheng Chen, Dengrui Fu, Wencai Ye, Dongmei Zhang

**Affiliations:** 1 College of Pharmacy, Jinan University, 510632 Guangzhou, China; 2 Guangdong Province Key Laboratory of Pharmacodynamic Constituents of Traditional Chinese Medicine and New Drugs Research, Jinan University, 510632 Guangzhou, China; 3 College of Pharmacy and Health Sciences, St. John’s University, Jamaica, NY 11439 USA; 4 Guangzhou Yucai Middle School, 510080 Guangzhou, China

## Abstract

Betulinic acid (BA) and its derivatives are a class of high-profile drug candidates, but their anticancer effects on resistant cancer have rarely been reported. Although a few studies indicated mitophagy is related with drug resistance, its role in different cancer types and anticancer agents treatment remains largely unclear. Here, we find that B5G1, a new derivative of BA, induces cell death in multidrug resistant cancer cells HepG2/ADM and MCF-7/ADR through mitochondrial-apoptosis pathway. B5G1 also triggers mitophagy independent on Atg5/Beclin 1. Further mechanistic study indicates that B5G1 upregulates PTEN-induced putative kinase 1 (PINK1) to recruit Parkin to mitochondria followed by ubiquitination of Mfn2 to initiate mitophagy. Inhibition of mitophagy by PINK1 siRNA, mdivi-1, or bafilomycin A1 (Baf A1) promotes B5G1-induced cell death. In addition, ROS production and mitochondrial damage in B5G1-treated HepG2/ADM cells cause mitochondrial apoptosis and mitophagy. In vivo study shown that B5G1 dramatically inhibits HepG2/ADM xenograft growth accompanied by apoptosis and mitophagy induction. Together, our results provide the first demonstration that B5G1, as a novel mitophagy inducer, has the potential to be developed into a drug candidate for treating multidrug resistant cancer.

## Introduction

Multidrug resistance (MDR) mediated by ATP-binding cassette (ABC) transporters is the primary obstacle to successful cancer chemotherapy^1^. Although numerous MDR reversal agents targeting ABC transporters have been developed, poor efficacy and severe side effects have caused their failure in clinical trials^2,3^. Therefore, the need to explore novel chemotherapeutic agents and effective strategies against resistant cancers is urgent.

Mitophagy is a type of selective autophagy that promotes mitochondrial turnover and prevents the accumulation of dysfunctional mitochondria to maintain cellular homeostasis. Recently, several reports suggested that mitophagy contribute to chemotherapeutic efficacy or drug resistance in cancer. In melanoma cells, inhibition of the mitochondrial respiratory chain by BAY 87-2243 induced mitophagy-dependent necroptosis and ferroptosis^4^. Targeting orphan nuclear receptor TR3 with a small molecule led to permeability transition pore opening, which results in excessive mitophagy and irreversible A375 cell death^5^. Selenite induced superoxide anion-mediated mitophagic cell death in glioma cells^6^. On the other hand, Doxorubicin (Dox)-induced mitophagy contributes to drug resistance in HCT8 human colorectal cancer stem cells. Inhibiting mitophagy by silencing BNIP3L enhanced Dox sensitivity in colorectal cancer stem cells^7^. Liensinine sensitized breast cancer cells to chemotherapy by mitophagy inhibition through DNM1L-mediated mitochondrial fission^8^. Although mitophagy is related with drug resistance, its role in different cancer types and anticancer agents treatment remains largely unclear.

Currently, a mechanism of mitophagy based on PTEN-induced putative kinase 1 (PINK1) and Parkin, an E3 ubiquitin ligase, is widely accepted. When mitochondrial membrane potential (MMP) is impaired by ROS, irradiation, or chemotherapeutic agents, PINK1 is stabilized on the outer mitochondrial membrane, leading to Parkin recruitment to damaged mitochondria^9^. Mitochondrial-anchored Parkin is phosphorylated at Ser65 by PINK1 and performs ubiquitination; this process results in further ubiquitination of other mitochondrial proteins, such as VDAC, TOM20, and Mfn2, to facilitate impaired mitochondria recognition^10^. However, Parkin-independent mitophagy has also been reported^11,12^. As a selective type of autophagy, the formation of mitochondrial autophagosomes is also subject to the regulatory mechanisms of autophagy. This process depends on autophagy-related proteins, such as Beclin 1, Atg5, and Atg12, for the formation, elongation, and closure of LC3-coated phagophores^13^. However, the roles of autophagy regulatory proteins differ in various types of cancers, and their underlying mechanisms are complicated and not fully understood. Therefore, the discovery of small molecule probes modulating mitophagy will be highly significant for revealing the molecular mechanisms of mitophagy.

Natural products and their derivatives are primary sources of anticancer agents that act via novel mechanisms. Betulinic acid (BA) and its derivatives, a class of high-profile bioactive agents, exhibit broad-spectrum anticancer activities, but little attention has been paid to their effects on multidrug-resistant cancer^14–17^. Accumulating evidence demonstrates that the mechanisms underlying cell death induced by BA and its derivatives are complicated and dependent on the cancer cell type. These compounds induce apoptosis in multiple myeloma, prostate cancer, and cervical cancer cells via multiple signaling pathways, such as the STAT3, NF-κB, and PI3K/Akt pathways^18–20^. Recent several studies have shown that BA and B10, a glycosylated derivative of BA, induce cell death by inhibiting autophagic flux in microglia, glioblastoma, and multiple myeloma cells^21–23^. In contrast, a few studies have reported that BA-induced autophagy as a pro-survival mechanism in colorectal, cervical, and breast cancer cells^24,25^. This pro-survival mechanism has been associated with p53 or the opening of the mitochondrial permeability transition pore^24^. However, the role of mitophagy has still not been investigated in cancer cells treated with BA or its derivatives.

In this study, we found that a new derivative of BA, B5G1, had potent anticancer activity towards multidrug-resistant cancer cells HepG2/ADM and MCF-7/ADR. B5G1 induced ROS production and mitochondrial dysfunction, thereby triggering mitophagy in a manner dependent on PINK1 and Parkin but not Atg5 and Beclin 1, and mitophagy inhibition promotes B5G1-induced apoptosis in drug-resistant cancer cells.

## Results

### B5G1 inhibits the proliferation of multidrug-resistant cancer cells via induction of mitochondrial apoptosis

**Fig. 1:**
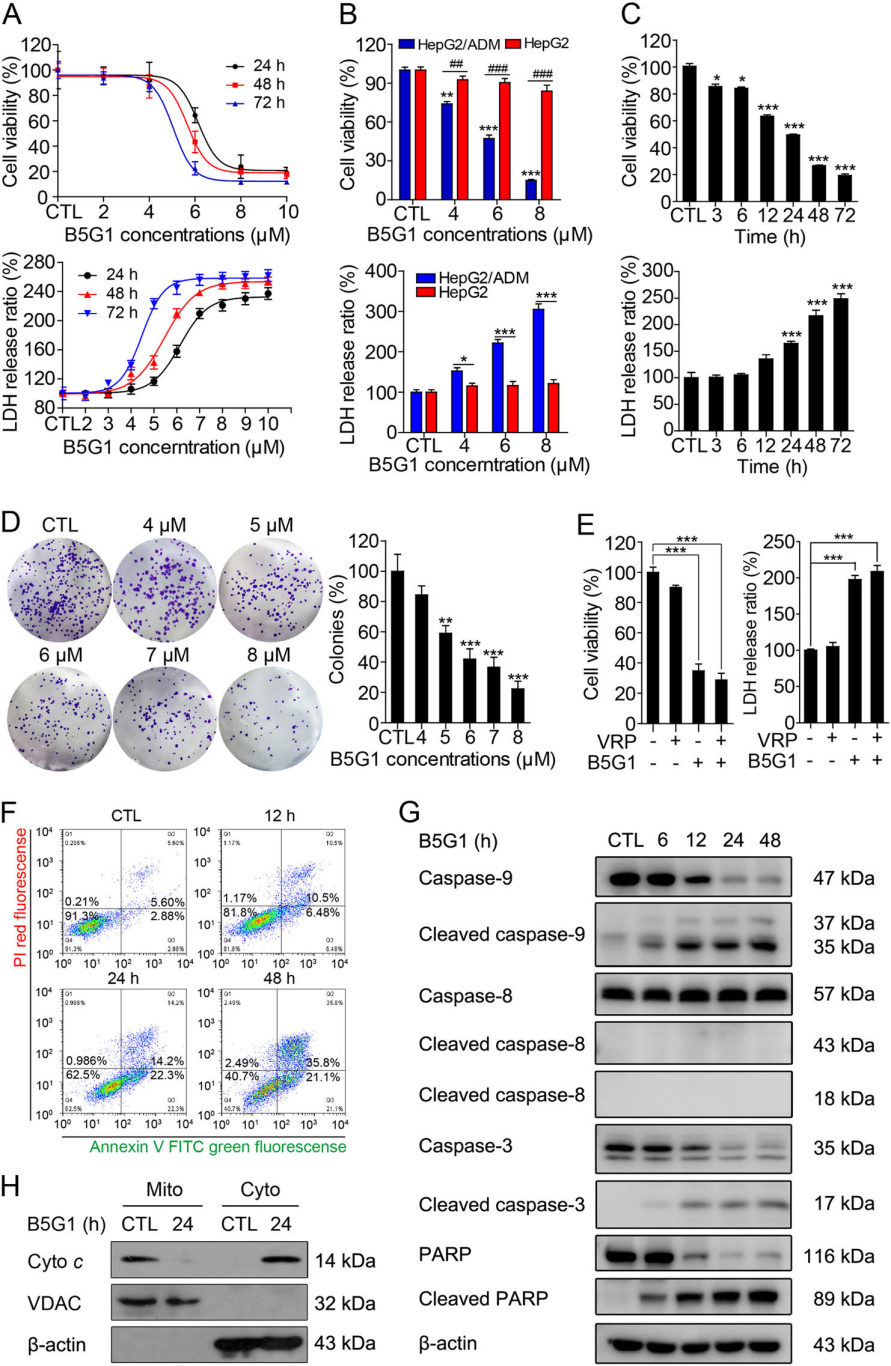
B5G1 inhibits the proliferation of HepG2/ADM cells *via* induction of mitochondrial apoptosis. **a** HepG2/ADM cells were treated with different concentrations of B5G1 for 24, 48, and 72 h. Cell viability was determined by MTT and LDH assay. **b** HepG2/ADM and HepG2 cells were treated with the indicated concentrations of B5G1 for 48 h, and cell viability was determined by MTT and LDH assay (*n* = 3). ^**^P *<* 0.01, ^***^P *<* 0.001 vs CTL (HepG2/ADM), ^##^*P* *<* 0.01, ^###^*P* *<* 0.001 (MTT assay); ^*^P *<* 0.05, ^***^P *<* 0.001 (LDH assay). **c** HepG2/ADM cells were treated with B5G1 (6 μM) for the indicated times, and cell viability was determined by MTT and LDH assay (*n* = 3). ^*^P *<* 0.05, ^***^P *<* 0.001 vs CTL. **d** HepG2/ADM cells were treated with the indicated concentrations of B5G1 for 24 h. Colonies were visualized by crystal violet staining and counted manually (*n* = 3). Magnification: ×200; ^**^P *<* 0.01, ^***^P *<* 0.001 vs CTL. **e** HepG2/ADM cells were treated with B5G1 (6 µM) in the presence or absence of VRP (50 µM) for 48 h, cell viability was measured by MTT and LDH assay (*n* = 3). ^***^P *<* 0.001. **f** The apoptosis rates of HepG2/ADM cells treated with B5G1 (6 μM) were detected by flow cytometry. **g** Apoptosis-related proteins expression level of HepG2/ADM cells treated with B5G1 (6 μM) for the indicated times were analyzed by western blotting. β-actin was used as a loading control. **h** Cell lysates of HepG2/ADM cells treated with B5G1 (6 μM) for 24 h were divided into cytoplasmic fractions and mitochondrial fractions. Cyto *c* translocation was measured by western blotting. β-actin and VDAC were used as loading controls for cytoplasm and mitochondria, respectively

B5G1 cytotoxicity against HepG2, HepG2/ADM, MCF-7, and MCF-7/ADR cells was evaluated by MTT assay and LDH assay. B5G1 showed selective cytotoxicity towards multidrug-resistant cancer cells HepG2/ADM and MCF-7/ADR but not their parent cells HepG2 and MCF-7 (Fig. 1a, b; Supplementary Fig. S1B and C). B5G1 decreased HepG2/ADM cell survival in a concentration- and time-dependent manner (Fig. 1a, c). It also inhibited the proliferation of HepG2/ADM colonies (Fig. 1d). HepG2/ADM is a multidrug-resistant cell line overexpressing ABCB1 (Supplementary Fig. S1D). ABCB1 substrates, such as Dox and vincristine (VCR), showed little cytotoxicity against HepG2/ADM cells. Determining whether B5G1 is a substrate of drug-resistant cells is important. As shown in Fig. 1e, VRP, a specific inhibitor of ABCB1, had no effect on B5G1-induced cell death, indicating that B5G1 is not a substrate of ABCB1.

Apoptosis induction is frequently reported in some cancer cell lines treated with BA or its derivatives. Similarly, the apoptotic rates increased in a time-dependent manner upon B5G1 treatment in HepG2/ADM (Fig. 1f) and MCF-7/ADR cells (Supplementary Fig. S1E). Caspase-9, caspase-3, and PARP cleavage was increased significantly after B5G1 treatment, but there was no significant change in caspase-8, a key mediator of the death receptor apoptosis pathway in HepG2/ADM (Fig. 1g) and MCF-7/ADR cells (Supplementary Fig. S1F). Moreover, cyto *c* was released from the mitochondria into the cytoplasm after B5G1 treatment (Fig. 1h; Supplementary Fig. S1G). Taken together, these results indicate that B5G1 induces multidrug-resistant cancer cell death via a mitochondrial apoptotic pathway.

### B5G1 induces mitophagy in multidrug-resistant cancer cells

**Fig. 2:**
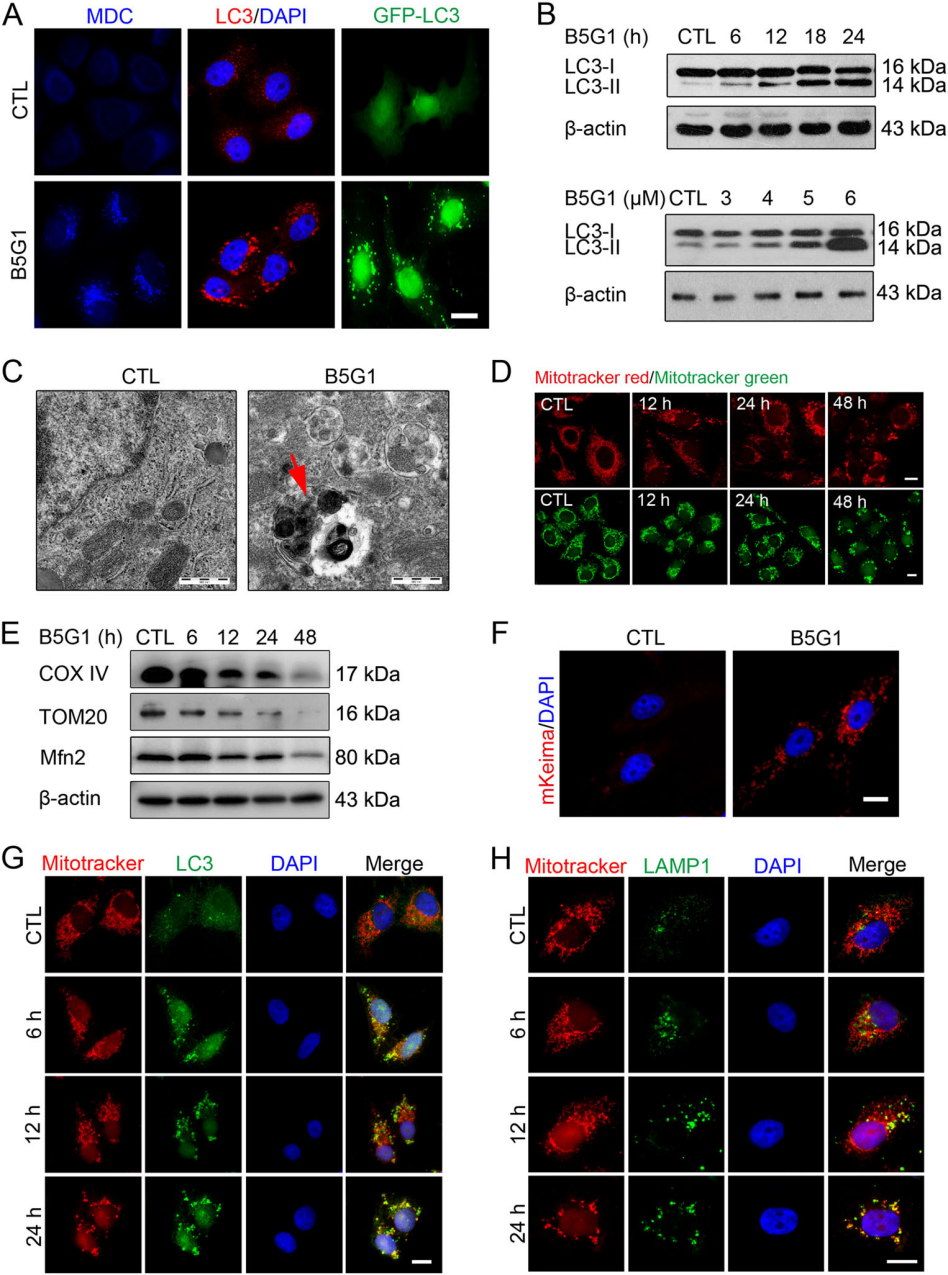
B5G1 induces mitophagy in HepG2/ADM cells. **a** After B5G1 treatment (6 μM) for 24 h, HepG2/ADM cells were stained with MDC (50 μM) or immunostained with a LC3 antibody. For the GFP-LC3 assay, HepG2/ADM cells were transfected with the GFP-LC3 plasmid for 24 h, followed by treatment with B5G1 (6 μM) for 24 h. The fluorescence was observed by a fluorescence microscope. Magnification: ×630, scale bar: 10 μm. **b** HepG2/ADM cells were treated with B5G1 as indicated, and LC3 expression level was then detected by western blotting. β-actin was used as a loading control. **c** After treatment with B5G1 (6 μM) for 24 h, the ultrastructure of HepG2/ADM cells was observed by a transmission electron microscopy. Magnification: ×8900; Scale bar: 500 nm. **d** HepG2/ADM cells were treated with B5G1 (6 μM) for 0, 12, 24, or 48 h and then stained with MitoTracker Red (200 nM) or MitoTracker Green (200 nM). The fluorescence was observed by a fluorescence microscope. Magnification: ×630; scale bar: 10 μm. **e** HepG2/ADM cells were exposed to B5G1 (6 μM) for the indicated times. Mitochondrial proteins expression levels were measured by western blotting. β-actin was used as a loading control. **f** HepG2/ADM cells were transfected with the mKeima-Red-Mito-7 plasmid, followed by treatment with B5G1 (6 μM) for 24 h. The Fluorescence was detected by a fluorescence microscope. Magnification: ×630; scale bar: 10 μm. **g**, **h** After treatment with B5G1 (6 μM) for the indicated times, HepG2/ADM cells were stained with MitoTracker red (200 nM) and immunostained with a LC3 or LAMP1 antibody. Mitochondrial colocalization with LC3 or LAMP1 was observed by a fluorescence microscope. Magnification: ×630; scale bar: 10 μm

As BA-induced autophagy in several cancer lines, we speculated that B5G1 would also induce autophagy in drug-resistant cancer cells. As expected, after B5G1 treatment for 12 h, MDC-positive autophagic vesicles were present in HepG2/ADM cells (Fig. 2a), and LC3 accumulation was found (Fig. 2a, b). Moreover, upon B5G1 treatment, bilayer membrane-bound autophagosomes were observed by transmission electron microscopy, further indicating that the induction of autophagy is mediated by B5G1 (Fig. 2c).

Both MitoTracker red and Mitotracker green staining showed that the number of mitochondria decreased gradually (Fig. 2d), and the mitochondrial proteins COX IV, TOM20, and Mfn2 were degraded in a time-dependent manner in HepG2/ADM cells (Fig. 2e), as well as MCF-7/ADR cells which were inhibited by Baf A1 (Supplementary Fig. S2A and B). These data indicated that mitochondrial proteins are likely to be eliminated by mitophagy rather than inhibition of de nove protein synthesis. To further confirm the occurrence of mitophagy, we transfected HepG2/ADM cells with mito-Keima plasmids. Mito-Keima is a pH-sensitive fluorescent protein located in mitochondria that can be used to identify mitochondrial movement from the cytoplasm (green) to lysosomes (red). As shown in Fig. 2f, red spots appeared in the cytoplasm after B5G1 treatment, indicating that mitochondria tended to form autolysosomes. In addition, the colocalization of mitochondria with LC3 or LAMP1 (a lysosome marker) further demonstrated mitophagy induction by B5G1 in HepG2/ADM and MCF-7/ADR cells (Fig. 2g, h; Supplementary Fig. S2C and D).

### B5G1-induced mitophagy is regulated by a nonclassical autophagy pathway

**Fig. 3:**
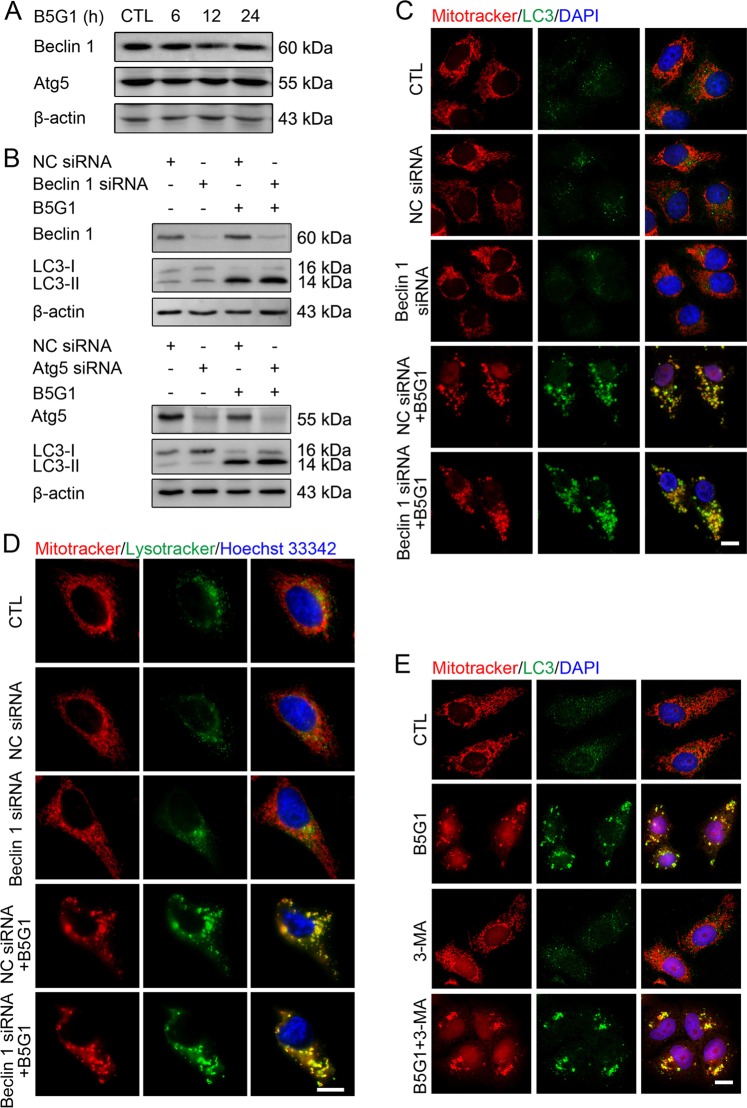
B5G1 induces mitophagy through a nonclassical autophagy pathway. **a** Autophagy-related proteins expression level of HepG2/ADM cells treated with B5G1 (6 µM) for the indicated times was analyzed by Western blotting. β-actin was used as a loading control. **b** HepG2/ADM cells were pretreated with NC siRNA, Beclin 1, or Atg5 siRNA for 24 h, followed by treatment with B5G1 (6 µM) for 12 h. Beclin 1, Atg5, and LC3 expression levels were determined by western blotting. β-actin was used as a loading control. **c** HepG2/ADM cells pretreated with NC or Beclin 1 siRNA were exposed to B5G1 (6 µM) for 24 h, the cells were stained with MitoTracker red (200 nM) followed by immunostaining with a LC3 antibody. Mitochondrial colocalization with LC3 was detected by a fluorescence microscope. Magnification: ×630; scale bar: 10 μm. **d** HepG2/ADM cells pretreated with NC or Beclin 1 siRNA were exposed to B5G1 (6 µM) for 24 h, the cells were then stained with MitoTracker red (200 nM), LysoTracker green (100 nM) and Hoechst 33342 (2 μg/ml), and mitochondrial colocalization with lysosomes was detected by a fluorescence microscope. Magnification: ×630; scale bar: 10 μm. **e** HepG2/ADM cells pretreated with 3-MA (5 mM) for 1 h were exposed to B5G1 (6 µM) for 24 h, the cells were stained with MitoTracker red (200 nM) followed by immunostaining with a LC3 antibody. Mitochondrial colocalization with LC3 was detected by a fluorescence microscope. Magnification: ×630; scale bar: 10 μm

Because Beclin 1 and Atg5 are two well-known molecules involved in autophagy regulation^27^, we determined the influence of B5G1 on these two regulators. Notably, the expression levels of these two proteins showed no apparent change upon B5G1 treatment (Fig. 3a). Knocking down the expression of Atg5 or Beclin 1 had no influence on LC3-II levels (Fig. 3b). Furthermore, B5G1-induced colocalization of clustered mitochondria with LC3 or lysosomes was not affected by Beclin 1 siRNA pretreatment (Fig. 3c, d). Futhermore, 3-MA, a Class III PI3K inhibitor, also failed to abolish the colocalization of mitochondria with LC3 (Fig. 3e). These results implied that the mitophagy caused by B5G1 is regulated by a nonclassical pathway that is independent of Beclin 1 and Atg5.

### B5G1-induced mitophagy is mediated by PINK1/Parkin

**Fig. 4:**
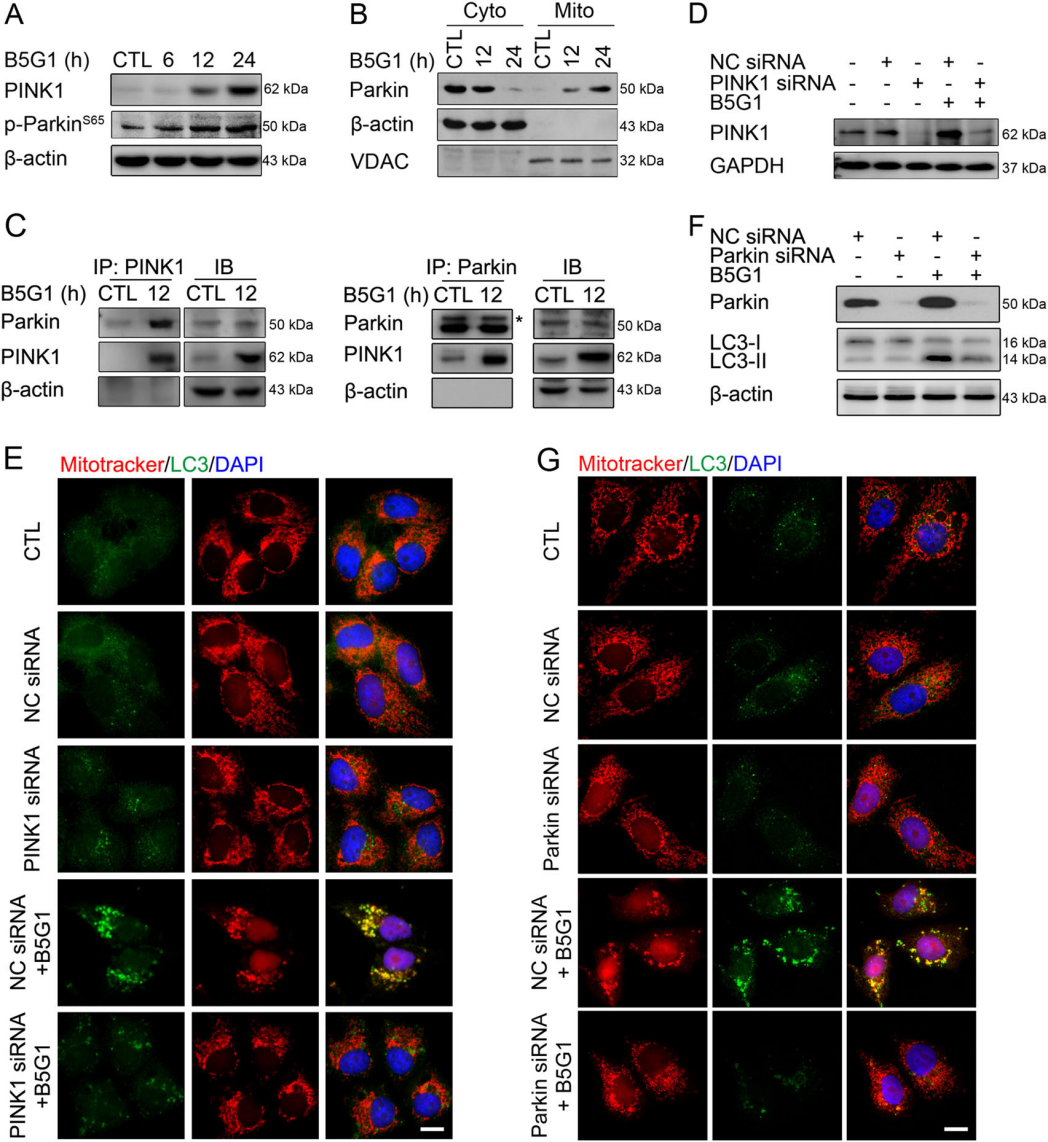
B5G1-induced mitophagy is mediated by the PINK1/Parkin pathway. **a** After treatment with B5G1 (6 µM) for the indicated times, PINK1 and p-Parkin (Ser65) expression levels were detected by western blotting. β-actin was used as a loading control. **b** HepG2/ADM cells treated with B5G1 (6 µM) for the indicated times were lysed and then separated into mitochondrial fractions and cytosolic fractions. Parkin translocation was measured by Western blotting. β-actin and VDAC were used as loading controls for the cytosolic and mitochondrial proteins, respectively. **c** After treatment with B5G1 (6 µM) for 12 h, HepG2/ADM cells were lysed with CO-IP lysis buffer, and the interaction between PINK1 and Parkin was measured by Co-IP assay. The asterisk indicated the band of Parkin. **d** HepG2/ADM cells were pretreated with NC or PINK1 siRNA, followed by treatment with B5G1 (6 µM) for 12 h. PINK1 expression levels were detected by western blotting. GAPDH was used as a loading control. **e** HepG2/ADM cells were transfected with NC or PINK1 siRNA for 24 h and then treated with B5G1 (6 µM) for another 24 h. Mitochondrial colocalization with LC3 was observed by a fluorescence microscope. Magnification: ×630; scale bar: 10 μm. **f** HepG2/ADM cells were transfected with NC or Parkin siRNA for 24 h and then treated with B5G1 (6 µM) for 12 h. Parkin and LC3 expression levels were detected by western blotting. β-actin was used as a loading control. **g** HepG2/ADM cells were transfected with NC or Parkin siRNA for 24 h and then treated with B5G1 (6 µM) for 24 h. Mitochondrial colocalization with LC3 was detected by immunofluorescence. Magnification: ×630; scale bar: 10 μm

PINK1 and Parkin are two key factors that regulate mitophagy. Parkin is translocated to the mitochondrial surface through the upregulation of PINK1, which in turn initiates mitophagy^28^. We found that PINK1 was significantly upregulated after B5G1 treatment in HepG2/ADM (Fig. 4a) and MCF-7/ADR cells (Supplementary Fig. S2E). Parkin was translocated from the cytoplasm to the mitochondria, and p-Parkin (Ser65) was upregulated by B5G1 in HepG2/ADM (Fig. 4a, b) and MCF-7/ADR cells (Supplementary Fig. S2F). A coimmunoprecipitation assay was performed to further investigate the interaction between PINK1 and Parkin. As shown in Fig. 4c, levels of PINK1 and the Parkin complex increased upon B5G1 treatment for 12 h in HepG2/ADM cells. These results indicated that B5G1-mediated mitophagy is associated with PINK1 and Parkin. To further confirm the role of PINK1 and Parkin in B5G1-induced mitophagy, siRNA-mediated PINK1 knockdown was conducted. In contrast to control siRNA, PINK1 siRNA significantly abolished mitochondrial colocalization with LC3 in HepG2/ADM cells (Fig. 4d, e). Knockdown of Parkin also get the same effect (Fig. 4f, g). Taken together, these findings indicate that B5G1 induces mitochondrial PINK1 upregulation to recruit Parkin, thereby initiating mitophagy.

### B5G1 induces Mfn2 ubiquitylation and mitochondrial anchoring of the autophagy adaptor p62/SQSTM1

**Fig. 5:**
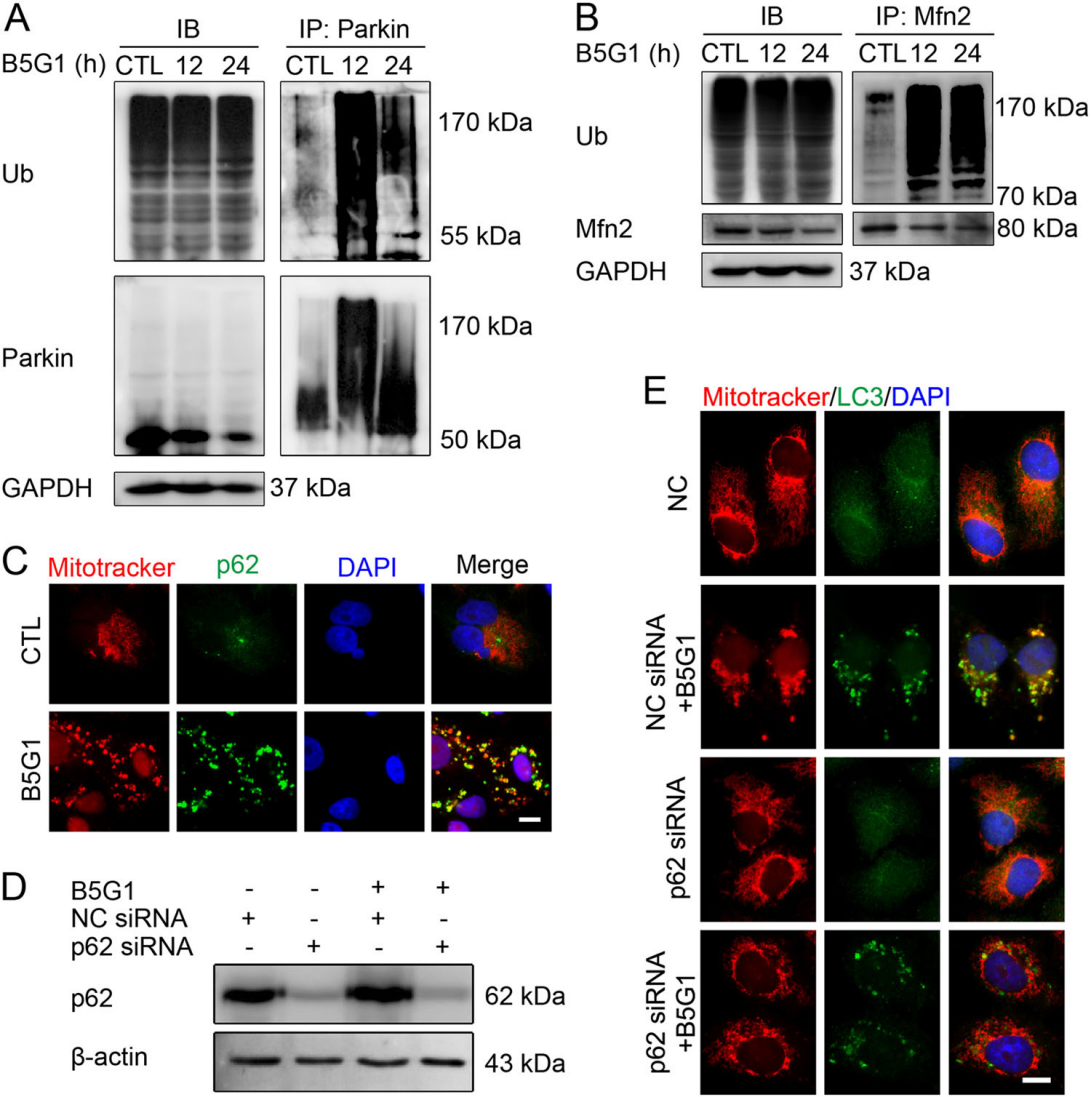
B5G1 induces Mfn2 ubiquitination and mitochondrial anchoring of the autophagy adaptor p62/SQSTM1. **a**, **b** HepG2/ADM cells were treated with B5G1 (6 µM) for the indicated times, Parkin and Mfn2 ubiquitination were detected by coimmunoprecipitation. **c** HepG2/ADM cells were treated with B5G1 (6 µM) for 24 h, and mitochondrial colocalization with p62 was detected by immunofluorescence. Magnification: ×630; scale bar: 10 μm. **d** HepG2/ADM cells were transfected with NC or p62 siRNA before treatment with B5G1 (6 µM) for 12 h. p62 expression level was detected by western blotting. β-actin was used as a loading control. **e** HepG2/ADM cells were transfected with NC or p62 siRNA and then treated with B5G1 (6 µM) for 24 h. Mitochondrial colocalization with LC3 was detected by immunofluorescence. Magnification: ×630; scale bar: 10 μm

Ubiquitylation of mitochondrial proteins, such as VDAC, Mfn2, and TOM20, is required for the recognition of damaged mitochondria by autophagy-related proteins^10^. In this process, Parkin is ubiquitylated by PINK1 first, so we detected Parkin ubiquitylation by western blotting in HepG2/ADM cells. Upon B5G1 treatment, ubiquitin modification of Parkin was detected by CO-IP assays (Fig. 5a). Moreover, ubiquitin modification of Mfn2 (Fig. 5b), but not TOM20 and VDAC (data not shown), was detected after B5G1 treatment. The ubiquitin-binding protein p62/SQSTM1 targets ubiquitylated mitochondrial proteins and binds them to form the autophagosomes that act in mitophagy. To investigate whether p62 is associated with B5G1-induced mitophagy, we analyzed the cellular localization of p62. As shown in Fig. 5c, p62 showed significant colocalization with mitochondria after B5G1 treatment for 12 h, and p62 siRNA pretreatment abolished the colocalization of mitochondria and LC3 in HepG2/ADM cells (Fig. 5d, e). In summary, these data show that Mfn2 ubiquitylation and p62 recruitment to mitochondria take part in PINK1/Parkin-mediated mitophagy in B5G1-treated resistant cancer cells.

### Mitophagy inhibition counterbalances apoptosis and sensitizes multidrug-resistant cancer cells to B5G1

**Fig. 6:**
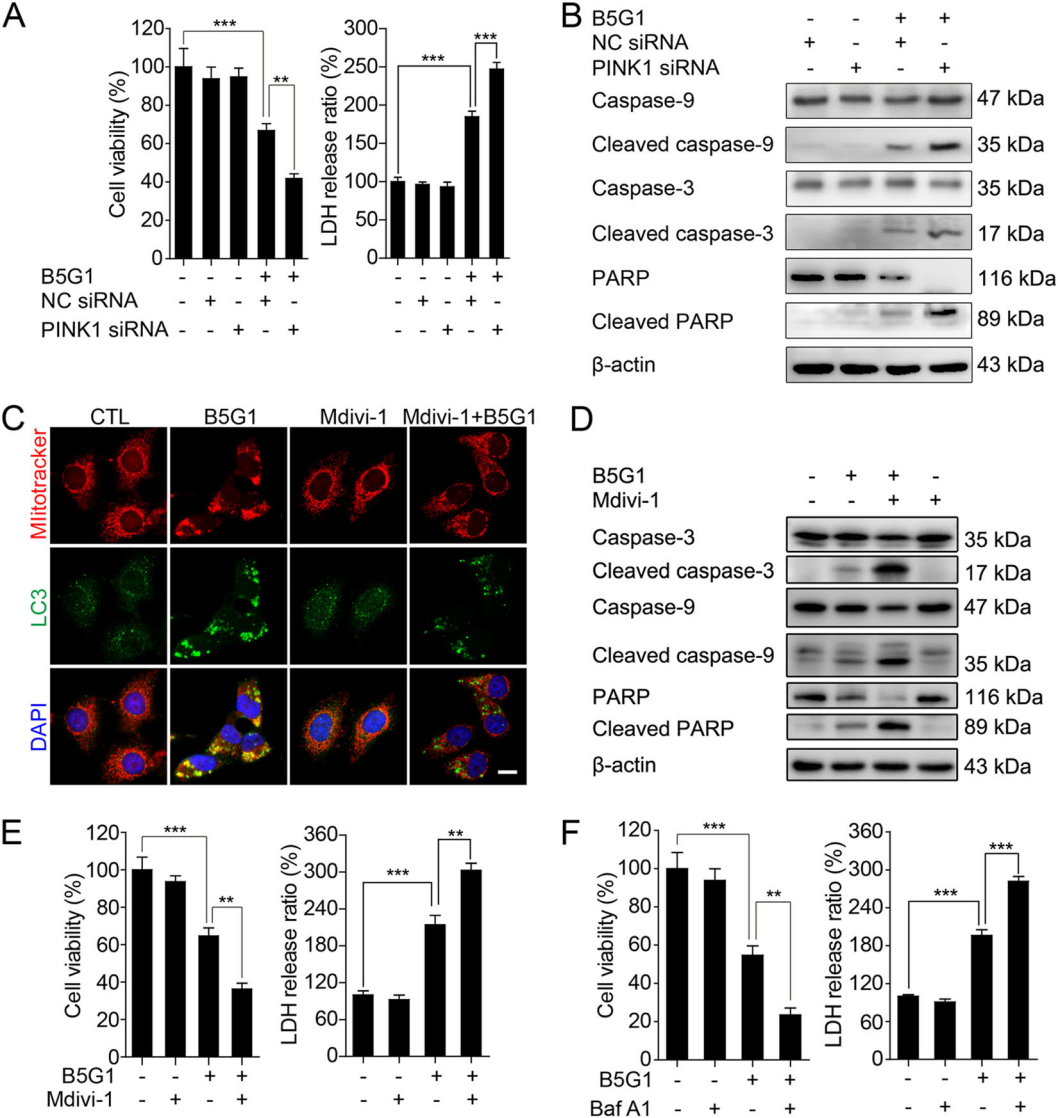
Inhibition of mitophagy sensitizes HepG2/ADM cells to B5G1 treatment. **a** HepG2/ADM cells were transfected with NC or PINK1 siRNA and then treated with B5G1 (5 µM) for 48 h. Cell viability was measured by MTT and LDH assay (*n* = 3). ^**^P *<* 0.01, ^***^P *<* 0.001. **b** Apoptosis-related proteins expression level in transfected cells was detected by western blotting after B5G1 (6 µM) treatment for 48 h. β-actin was used as a loading control. **c** HepG2/ADM cells were pretreated with mdivi-1 (10 µM) for 1 h, followed by treatment with B5G1 (6 µM) for another 24 h. Mitochondrial colocalization with LC3 was detected by immunofluorescence. Magnification: ×630; scale bar: 10 μm. **d** HepG2/ADM cells were pretreated with mdivi-1 (10 µM) for 1 h, followed by treatment with B5G1 (6 µM) for another 48 h. Apoptosis-related proteins expression level was detected by western blotting. β-actin was used as a loading control. **e** HepG2/ADM cells were pretreated with mdivi-1 (10 µM) for 1 h, followed by treatment with B5G1 (6 µM) for another 48 h. Cell viability was measured by MTT and LDH assay (*n* = 3). ^**^P *<* 0.01, ^***^P *<* 0.001. **f** HepG2/ADM cells were pretreated with Baf A1 (200 nM) for 1 h, followed by treatment with B5G1 (6 µM) for another 48 h. Cell viability was measured by MTT and LDH assay (*n* = 3). ^**^P *<* 0.01, ^***^P *<* 0.001

As mitophagy acts as “a double-edged sword” in cancer, we next investigated the interaction of B5G1-induced mitophagy and apoptosis in drug-resistant cancer cells using PINK1 siRNA, a mitophagy inhibitor, mdivi-1, and a lysosome inhibitor, Baf A1. PINK1 siRNA pretreatment increased B5G1-induced cell death as well as caspase-3, caspase-9, and PARP cleavage in HepG2/ADM cells (Fig. 6a, b). Mdivi-1, a mitophagy inhibitor^4,29^, was used to further investigate the role of B5G1-induced mitophagy. Pretreatment with mdivi-1 inhibited the B5G1-induced colocalization of mitochondria with LC3 (Fig. 6c). The corresponding apoptotic proteins were further activated in the presence of mdivi-1 in B5G1-treated HepG2/ADM cells (Fig. 6d). Furthermore, mdivi-1 pretreatment increased B5G1-induced apoptosis and cell death in HepG2/ADM cells (Fig. 6d, e). Baf A1, which inhibits the fusion of autophagosomes with lysosomes, also sensitized HepG2/ADM cells to B5G1 (Fig. 6f). In addition, PINK1 depletion and Baf A1 pretreatment also sensitized MCF-7/ADR cells to B5G1 treatment (Supplementary Fig. S3A-C). In conclusion, B5G1-induced mitophagy served a protective function in HepG2/ADM and MCF-7/ADR cells, and blocking mitophagy may enhance the therapeutic efficacy of B5G1 against drug-resistant cancer cells.

### B5G1 triggers mitochondrial ROS release to induce apoptosis and activate mitophagy

**Fig. 7:**
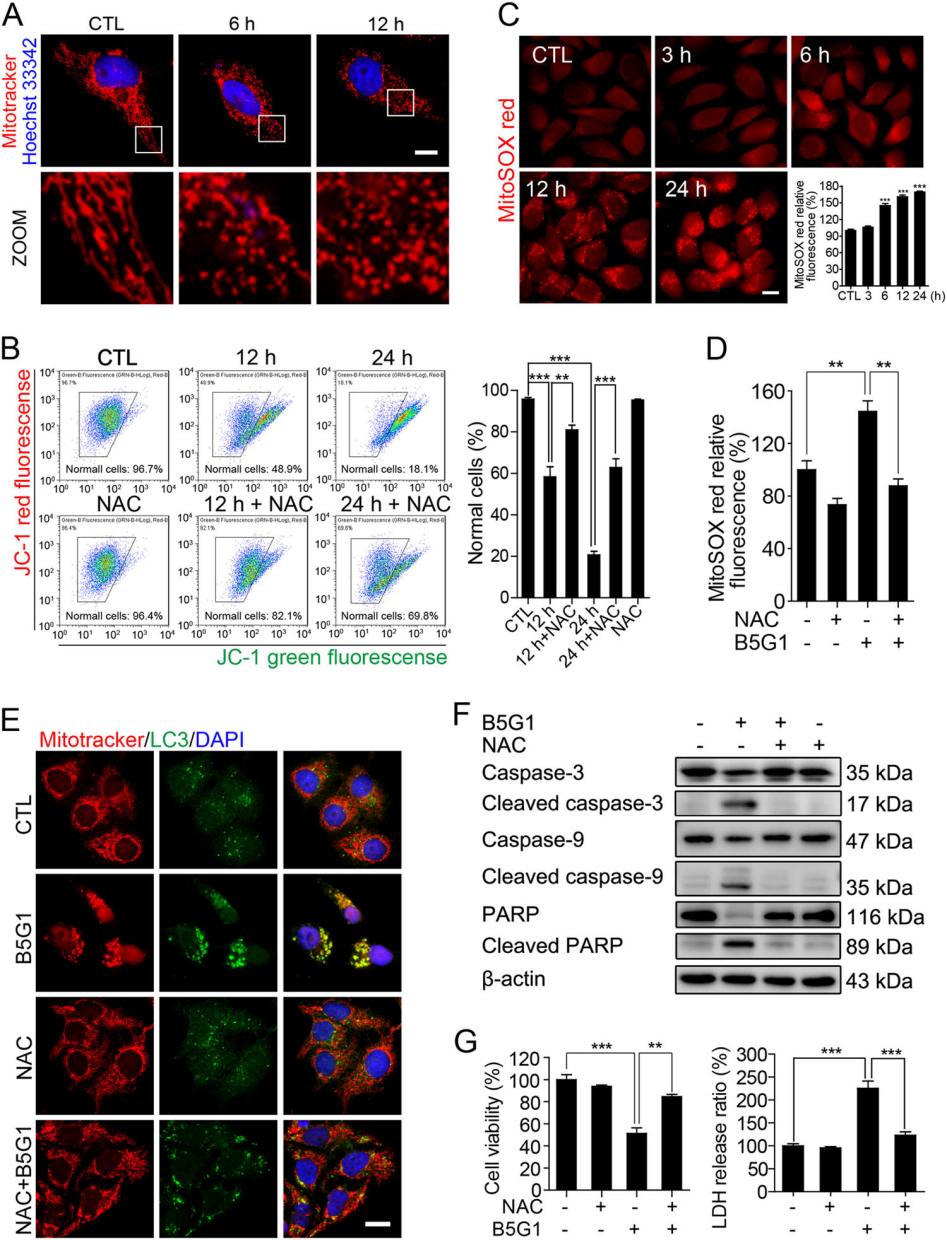
B5G1 activates mitophagy by inducing mitochondrial ROS overproduction. **a** After treatment with B5G1 (6 μM) for the indicated times, HepG2/ADM cells were stained with MitoTracker red (200 nM) and Hoechst 33342 (2 μg/ml), the mitochondrial morphology was observed by a fluorescence microscope. Magnification: ×630; scale bar: 10 μm. **b** HepG2/ADM cells were treated with B5G1 (6 μM) in the presence or absence of NAC (20 mM) for 12 and 24 h, changes in MMP were determined using JC-1 staining by flow cytometry analysis (*n* = 3). ^**^P *<* 0.01, ^***^P *<* 0.001. **c** HepG2/ADM cells were treated with B5G1 (6 μM) for the indicated times, and stained with MitoSOX red (10 μM), the fluorescence was observed by a fluorescence microscope or measured by a microplate reader. Magnification: ×630; scale bar: 10 μm (*n* = 3), ^***^P *<* 0.001 *vs* CTL. **d** HepG2/ADM cells were treated with B5G1 (6 μM) in the presence or absence of NAC (20 mM) for 6 h, and stained with MitoSOX red (10 μM), the fluorescence was measured by a microplate reader (*n* = 3). ^**^P *<* 0.01. **e** After treatment with B5G1 (6 μM) in the presence or absence of NAC (20 mM) for 24 h, HepG2/ADM cells were stained with MitoTracker red (200 nM) and immunostained with a LC3 antibody, mitochondrial colocalization with LC3 was observed by a fluorescence microscope. Magnification: ×630; scale bar: 10 μm. **f** HepG2/ADM cells treated with B5G1 (6 μM) for 48 h in the presence or absence of NAC (20 mM). Apoptosis-related proteins expression level were analyzed by western blotting. β-actin was used as a loading control. **g** HepG2/ADM cells treated with B5G1 (6 μM) for 48 h in the presence or absence of NAC (20 mM). Cell viability was determined by MTT and LDH assay (*n* = 3). ^**^P *<* 0.01, ^***^P *<* 0.001

Mitochondrial dysfunction usually results in mitophagy, which eliminates damaged mitochondria. We next aimed to study the cause of B5G1-induced mitophagy. MitoTracker staining assays were used to detect the mitochondrial morphology. As shown in Fig. 7a, filiform mitochondria were observed in untreated HepG2/ADM cells. In contrast, mitochondria in B5G1-treated HepG2/ADM cells were shrunken and fragmented into small units. Moreover, the MMP of HepG2/ADM cells significantly decreased after B5G1 treatment (Fig. 7b). All these data indicate mitochondrial dysfunction. Mitochondria serve as the main suppliers of ROS in mammalian cells, and excessive ROS levels lead to mitochondrial damage. We found that mitochondrial ROS levels increased dramatically after B5G1 treatment for 6 h and further increased at 12 h and 24 h, which was in accordance with the occurrence of mitophagy (Fig. 7c). To investigate whether mitochondrial ROS overproduction initiates mitophagy, NAC, an ROS scavenger, was used to study the role of ROS in mitophagy. Clearance of mitochondrial ROS by NAC (Fig. 7d) restored decreased MMP (Fig. 7b) and inhibited mitochondrial LC3 anchoring (Fig. 7e). The data above indicate that B5G1-induced mitophagy is mitochondrial ROS dependent. On the other hand, NAC pretreatment markedly inhibited B5G1-induced caspase-9, caspase-3, and PARP cleavage, as well as cell death (Fig. 7f, g). Therefore, the overproduction of mitochondrial ROS serves as either a mitophagy activator or an apoptosis inducer in B5G1-treated HepG2/ADM cells.

### B5G1 suppresses tumor growth in HepG2/ADM xenografts by inducing apoptosis and mitophagy

**Fig. 8:**
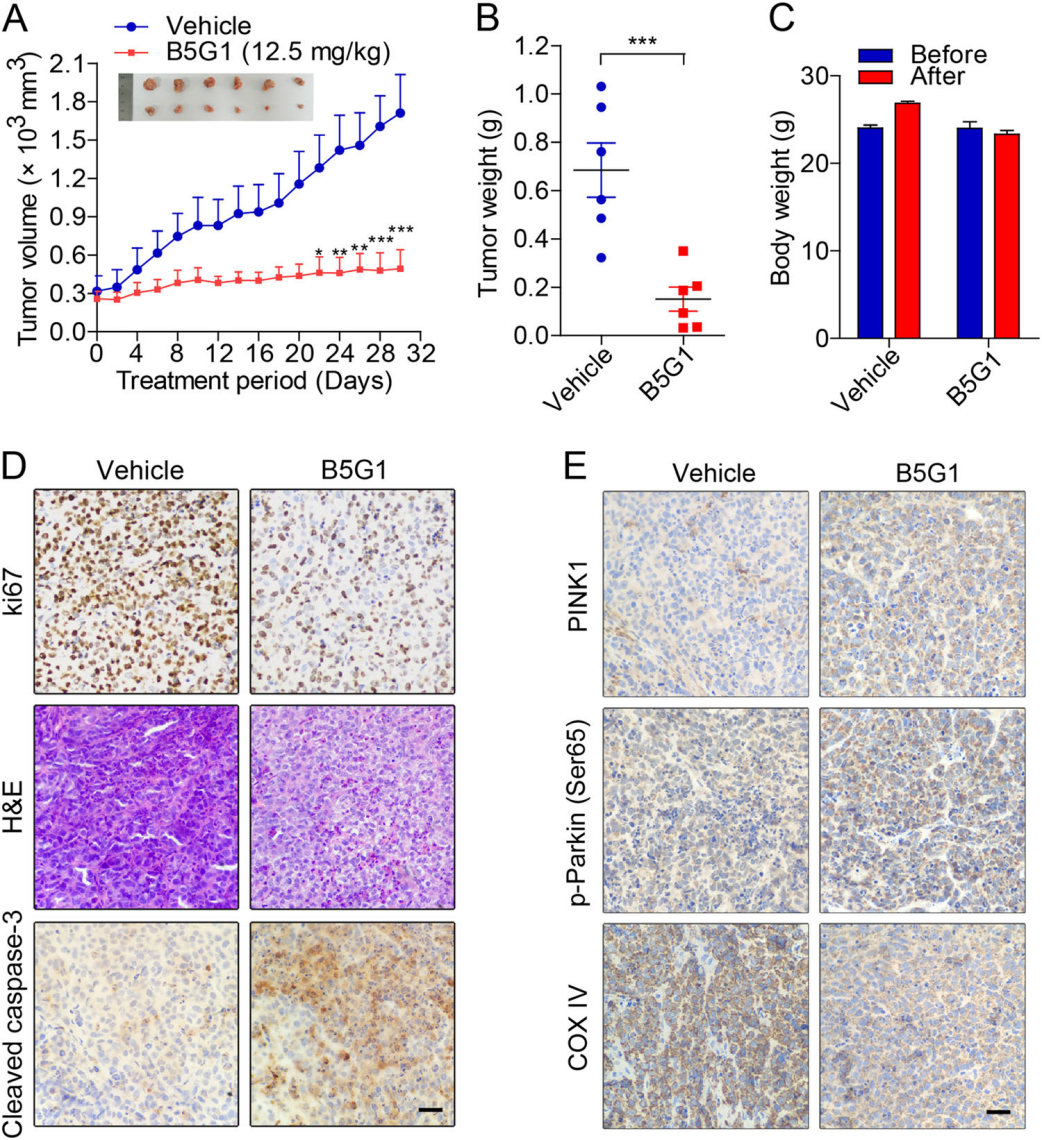
B5G1 suppresses tumor growth in a HepG2/ADM xenograft model. **a** Tumor volumes were measured every other day. At the end of the treatment period, the tumors were taken out and photographed (*n* = 6). ^*^P < 0.05, ^**^P < 0.01, ^***^P < 0.001 *vs* vehicle. **b** The finall tumors weight were measured. ^***^P < 0.001. **c** The mice body weight before and after B5G1 treatment. **d** The tumor tissues were subjected to H&E staining and immunohistochemistry staining for ki67 and cleaved caspase-3. Scale bar: 50 μm. **e** The tumor tissues were subjected to immunohistochemistry staining for PINK1, p-Parkin (Ser65), and COX IV. Scale bar: 50 μm.

To determine the in vivo therapeutic effects of B5G1 in multidrug-resistant cancer, HepG2/ADM cells were injected into immunodeficient nude mice treated with vehicle or B5G1 via intragastric administration once per day. As shown in Fig. 8a, B5G1 significantly suppressed the growth of HepG2/ADM xenografts. The final average tumor weight of the B5G1 group was 0.68 ± 0.28 g, which was much lower than that of the vehicle group (0.15 ± 0.12 g) (Fig. 8b). There were no significant differences in body weight between the two groups (Fig. 8c). H&E staining showed that B5G1 treatment caused dramatic cell death in the tumor sections, which could be a result of cellular proliferation inhibition and cancer cell apoptosis induction, as the B5G1 group had a lower ki67 index, a cellular marker for proliferation, and higher amount of cleaved caspase-3-positive cells than the vehicle group (Fig. 8d). Immunohistochemical analyses showed that B5G1 markedly increased PINK1 and p-Parkin (Ser65) expression levels and decreased COX IV expression levels (Fig. 8e), suggesting that B5G1 also activated mitophagy to clear damaged mitochondrial proteins. These data suggested that B5G1 inhibits tumor growth accompanied by apoptosis and mitophagy induction in vivo.

## Discussion

ABC transporter-mediated MDR is an important reason for cancer chemotherapy failure. The development of new anticancer agents that can overcome MDR has attracted much attention. Here, we report for the first time that B5G1, a derivative of BA, overcame multidrug-resistant cancer by inducing mitochondrial apoptosis and mitophagy. In addition, the combination of B5G1 with mitophagy inhibitors, such as mdivi-1 and bafilomycin A1, enhanced the anticancer activity of B5G1 against multidrug-resistant cancer. Our study indicates that B5G1 could be developed as a drug candidate for treating multidrug-resistant cancer.

Mitophagy-induced mitochondrial clearance is a response to mitochondrial injury. When mitochondrial damage occurs, cells initiate mitophagy or mitochondrial fission to clear damaged mitochondria to maintain mitochondrial homeostasis. However, if the mitochondrial damage is excessive, mitochondrial ROS overload, mitochondrial apoptotic protein release and caspase activation can occur and ultimately trigger apoptosis. In our study, B5G1-induced mitochondrial dysfunction was a result of mitochondrial ROS overload, as NAC restored MMP collapse. NAC pretreatment significantly inhibited mitophagy, indicating that B5G1-induced mitophagy is a response to mitochondrial damage in an attempt to maintain cell homeostasis and counterbalance apoptosis. Inhibiting mitophagy by PINK1 siRNA or mdivi-1 enhanced B5G1-induced apoptosis, implying that inhibiting mitophagy leads to enhanced mitochondrial ROS accumulation and mitochondrial apoptotic protein release, resulting in excessive cellular stress and eventually leading to increased apoptosis. This may explain why mitophagy inhibition increased B5G1-induced cell death. PINK1/Parkin pathway has been identified as a paradigmatic mechanism for mammalian mitophagy. In this pathway, mitochondrial membrane proteins serve as Parkin substrates to identify damaged mitochondria. To date, several mitochondrial substrates of Parkin have been identified, such as mitofusins (Mfn1 and Mfn2), translocase of the outer mitochondrial membrane complexes (TOM20, TOM40, and TOM70), mitochondrial Rho GTPases (MIRO 1 and MIRO 2), VDAC, and FIS1^30,31^. Here, we have identified Mfn2 as a mitochondrial substrate for Parkin. Mfn2 is a mitochondrial outer membrane protein that participates in mitochondrial fusion and contributes to mitochondrial network maintenance^32^. When mitophagy occurs, cells undergo mitochondrial fission to promote mitophagy by separating mitochondria into fragments and favoring autophagosome packaging^33^. B5G1-induced Mfn2 ubiquitylation may inhibit mitochondrial fusion and facilitate fission to reduce the refusion of healthy mitochondria with damaged mitochondria and finally promote damaged mitochondria clearance. Additionally, p62 recognizes ubiquitylated Mfn2 and recruits LC3 to form autophagosomes^10^. Indeed, we found that p62 was recruited to damaged mitochondria upon B5G1 treatment. However, this process can be equally mediated by the ubiquitylation of VDAC^34^. Notably, we did not detect this phenomenon after B5G1 treatment. Our data support the notion that of the multiple mitochondrial substrates of Parkin, one takes part in the regulation of mitophagy dependent on the stimulating factors and cancer types.

The formation of mitochondrial autophagosomes is also highly regulated by ATG genes, such as Beclin 1 and Atg5. Beclin 1 interacts with Atg14 and Vps34 to form a PI3K complex that leads to the formation of a phosphatidylinositol 3-phosphate (PtdIns3P)-rich membrane^35^. This is a critical step in the nucleation of the isolation membrane. However, Beclin 1-independent autophagy and mitophagy have also been reported^36,37^. These findings may imply the existence of alternative pathways that mediate the formation of PtdIns3P. For example, the activation of phosphatases to remove the D4 phosphate from PtdIns3,4 P or the inhibition of phosphatases participating in removing the D3 phosphate from PtdIns3P could lead to PtdIns3P accumulation^38,39^. Wortmannin-resistant class II PI3Ks also produce PtdIns3P^40^. Moreover, a recent study showed that lipids synthesized in ER also involve in mitophagy, while mitochondria tether to ER to form mitophagosomes^41^. Whether these mechanisms are involved in B5G1-induced mitophagy requires further investigation. Nevertheless, our study supports the existence of an alternative pathway for PtdIns3P production or a PtdIns3P-independent pathway during mitophagy. However, non-canonical mitophagy induced by chemical stimulates is rarely reported and the underlying mechanism is largely unclear. Therefore, our work may contribute to arouse the interest in studying the mechanism of non-canonical mitophagy and discovering new mitophagy-related therapeutic targets to conquer multidrug-resistant cancer.

Accumulating evidence shows that autophagy plays dual roles (cytoprotective or cytotoxic) in response to cancer chemotherapy. The induction of autophagy by BA and its derivatives has been reported in several types of cancer cells. BA induces protective autophagy by inhibiting Akt/mTOR signaling in human colorectal cancer cells. Knocking down p53 attenuates autophagy and augmentes BA-induced cell death^24^. It has also been reported that autophagy activation in BA-treated MCF-7 and HeLa cells is abolished by CsA, indicating that BA-induced autophagy may be a consequence of mitochondrial damage. Inhibiting autophagy by Atg5 or Atg7 shRNA also enhances BA cytotoxicity^25^. B10, a glycosylated derivative of BA, induces autophagy in U87MG cells. However, the concomitant impairment of autophagic flux by B10 converts autophagy into a cell death mechanism^23^. Moreover, BA induces autophagic cell death in bladder cancer by downregulating phosphorylated Akt and degrading EGFR^42^. In our study, we first report that B5G1 induced PINK1/Parkin-mediated mitophagy via a mitochondrial ROS burst to antagonize mitochondrial apoptosis. However, we did not found BA had the similar effect in HepG2/ADM and MCF-7/ADR cells. Therefore, the role of mitophagy induced by BA and its derivatives dependents on cell types and chemical structures. Our works support that mitophagy could also act as a cytoprotective role in multidrug-resistant cancer cells treated with derivatives of BA which further indicating the complicated role of mitophagy in cancer cells.

In conclusion, our study indicates that the BA analog B5G1 induces mitochondrial damage to initiate nonclassical Atg5/Beclin 1-independent but PINK1/Parkin-dependent mitophagy. Blocking mitophagy sensitizes multidrug-resistant cancer cells to B5G1. These novel properties of B5G1 are expected to have important implications for the development of therapeutic agents for multidrug-resistant cancer and the study of non-canonical mitophagy.

## Materials and methods

### Reagents and antibodies

B5G1 was synthesized from 23-hydroxybetulinic acid as described previously and had a purity of 98% (Supplementary Fig. S1A)^26^. [3-(4,5-dimethyl-2-thiazolyl)-2,5-diphenyl-2H-tetrazolium bromide] (MTT), verapamil (VRP), N-acetyl-cysteine (NAC), monodansylcadaverine (MDC), DAPI, DMSO, NP-40, Triton X-100, paraformaldehyde (PFA), HS 15, and Tween-20 were purchased from Sigma-Aldrich (St. Louis, MO, USA). Hoechst 33342, JC-1, MitoTracker® Red CMXRos, MitoTracker™ Green FM, MitoSOX Red and LysoTracker™ Green DND-26, as well as a BCA protein assay kit, a LDH assay kit, a Dead Cell Apoptosis kit, and a DAB kit were obtained from Thermo Fisher Scientific (Waltham, MA, USA). Dox and bafilomycin A1 (Baf A1) were purchased from Selleck (Houston, TX, USA). Mdivi-1 was obtained from MedChem Express (New Jersey, USA). Laemmli sample buffer (2×) and an ECL chemiluminescence detection kit were purchased from Bio-Rad (Hercules, CA, USA). Matrigel was obtained from BD Biosciences (San Jose, CA, USA). Antibodies against caspase-3, cleaved caspase-3, caspase-9, cleaved caspase-9, caspase-8, cleaved caspase-8, PARP, cleaved PARP, cytochrome *c* (cyto *c*), LC3, VDAC, Atg5, Beclin 1, Parkin, PINK1, p62, LAMP1, ubiquitin, ki67, β-actin, anti-rabbit IgG, and anti-mouse IgG were obtained from Cell Signaling Technology (Beverly, MA, USA). Antibodies against p-Parkin (Ser65) were obtained from Biorbyt-Biotechnology Company (Cambridge, Cambridgeshire, UK). A GFP-LC3 plasmid (#22405) and mKeima-Red-Mito-7 plasmid (#56018) were obtained from Addgene (Cambridge, MA, USA). All siRNAs were synthesized by GenePharma Co., Ltd. (Shanghai, China).

### Cell culture

HepG2/ADM cells, generously provided by Prof. Kwok-Pui Fung (Chinese University of Hong Kong, Hong Kong, China), and MCF-7/ADR cells, kindly donated by Prof. Liwu Fu (Sun Yat-Sen University, Guangzhou, China), were cultured in RPMI 1640 medium (Thermo Fisher Scientific, Waltham, MA, USA) supplemented with 10% (v/v) fetal bovine serum (Thermo Fisher Scientific), 1% (v/v) penicillin-streptomycin (PS; 10,000 U/ml, Thermo Fisher Scientific), and 1.2 µM Dox at 37 °C in a humidified atmosphere of 5% CO_2_. For the B5G1 treatment experiments, HepG2/ADM and MCF-7/ADR cells were cultured in complete medium without Dox.

### Cell viability assay

Cells (6.0 × 10^3^/well) were seeded in 96-well plates and cultured overnight. After treatment for the indicated times, 30 µl of MTT (5 mg/ml) was added to each well and incubated at 37 °C for an additional 4 h. The formazan crystals were solubilized in 100 µl of DMSO, and the absorbance was measured at 595 nm using a microplate reader (Beckman Coulter, Brea, CA, USA). Cell viability was calculated as a percentage of the vehicle control group treated with medium containing 0.2% DMSO.

### LDH assay

Cells (6.0 × 10^3^/well) were seeded in 96-well plates and cultured overnight. After treatment as indicated, cellular cytotoxicity was measured by detection of LDH release using a LDH assay kit according to the manufacturer’s protocol. The cellular cytotoxicity was calculated as the percentage of the ratio of LDH release.

### Colony formation assay

HepG2/ADM cells (2.5 × 10^5^/well) were seeded in 6-well plates and cultured overnight. The cells were then exposed to the indicated concentrations of B5G1 for 24 h and collected by trypsinization. Next, 600 cells/well were seeded in new 6-well plates and cultured for 10 days. On the 10th day, the cells were fixed with 4% paraformaldehyde and stained with a 0.1% crystal violet solution. The cell colonies were photographed using a CKX41 inverted microscope (Olympus, Japan) and counted manually.

### Apoptosis detection by flow cytometry

After B5G1 treatment for the indicated times, cells were collected and stained with an Annexin-V-FITC/PI staining assay kit according to the manufacturer’s protocol. The cell apoptotic rates were examined by a Guava Easy Cyte™ flow cytometer (Guava Technologies, Millipore, Billerica, MA, USA). The data were analyzed quantitatively using Flow Jo 7.6 software (TreeStar, San Carlos, CA, USA).

### Cell staining assay

Cells (1.5 × 10^5^/well) were seeded in 35-mm culture dishes and cultured overnight. After the indicated treatments, the cells were stained with the corresponding reagents according to different experimental purposes. Cells were incubated with MDC (50 μM) for 30 min at 37 °C in the dark to determine autolysosome formation. Cells were incubated with MitoTracker® Red CMXRos (200 nM) or Mitotracker Green (200 nM) for 30 min at 37 °C in the dark to detect mitochondrial morphology. Cells were treated with LysoTracker™ Green DND-26 (100 nM), MitoTracker® Red CMXRos (200 nM) and Hoechst 33342 (2 μg/ml) for 30 min at 37 °C in the dark to observe mitochondrion-lysosome fusion. Cell images were acquired using a Zeiss AX10 microscope (Carl Zeiss, Göttingen, Germany) (Ex = 335 nm, Em = 518 nm for MDC; Ex = 579 nm, Em = 599 nm for MitoTracker® Red CMXRos; Ex = 504 nm, Em = 511 nm for LysoTracker™ Green DND-26).

### Transmission electron microscopy assay

Cells were fixed with 4% glutaraldehyde overnight at 4 °C, followed by fixation with 1% osmium tetroxide for 1 h. Next, different concentrations of ethanol and acetone were used in sequence to dehydrate the cells. Then, the cells were polymerized with epoxy resin and cut into ultrathin sections. After staining with aqueous uranyl acetate and lead citrate, the ultrastructure of the cells was observed by a TECNAI 10 transmission electron microscope (Philips, Holland).

### Immunofluorescence assay

Cells growing on glass coverslips were exposed to the indicated treatments. The cells were fixed with 4% PFA and blocked with 5% BSA containing 0.4% Triton X-100. Then, the cells were incubated with a primary antibody against cyto *c*, LC3, LAMP1, and p62 at 4 °C overnight and a fluorescent secondary antibody for 1 h at room temperature and then stained with DAPI for 5 min. Finally, the cellular fluorescence was observed by a Zeiss AX10 microscope.

### GFP-LC3 plasmid transient transfection

HepG2/ADM cells were transfected with the GFP-LC3 plasmid using Lipofectamine 3000 according to the manufacturer’s instructions. After transfection, the HepG2/ADM cells were treated with B5G1 (5 μM) for the indicated times and fixed with 4% PFA. Images were captured by a Zeiss AX10 microscope to observe autophagosome accumulation.

### Mito-Keima mitophagy analysis

HepG2/ADM cells were transfected with the mKeima-Red-Mito-7 plasmid using Lipofectamine 3000 for 24 h and then treated with B5G1 for another 24 h. The cells were imaged using a Zeiss AX10 microscope (Ex = 550 nm, Em = 620 for acidic red fluorescence).

### Small interfering RNA (siRNA) transfection assay

Cells were transfected with scrambled siRNA duplexes or specific siRNA duplexes targeting Beclin 1, Atg5, Parkin, PINK1, and p62 using Lipofectamine 3000 according to the manufacturer’s protocol. After transfection, the cells were treated with B5G1 for the indicated times, and the expression levels of these proteins were analyzed by western blotting. The scrambled siRNA duplexes were regarded as negative controls with nontargeting sequences. The sequences of the siRNAs were as

follows: Atg5: 5′-GACAAGAAGACAUUAGUGA-3′; Beclin 1: 5′-GGUCUAAGACGUCCAACAATT-3′; PINK1: 5′-CGCUGUUCCUCGUUAUGAATT-3′; Parkin: 5′-GCCACGUGAUUUGCUUAGATT-3′; p62: 5′-GUGACGAGGAAUUGACAAUTT-3′; NC: 5′-UUCUCCGAACGUGUCACGUTT-3′.

### MMP assay

MMP was detected by JC-1 staining assays. After treatment with B5G1 for the indicated times, HepG2/ADM cells were collected by trypsinization and stained with JC-1 (5 μM) for 15 min at 37 °C in the dark. MMP was detected by a Guava Easy Cyte™ flow cytometer (Ex = 488 nm and Em = 590 nm for JC-1 aggregates; Ex = 488 nm and Em = 529 nm for JC-1 monomers).

### Mitochondrial ROS measurement

HepG2/ADM cells were seeded in 35-mm dishes and treated with B5G1 (5 µM) for the indicated times. Then, the cells were exposed to MitoSOX Red (10 µM) for 30 min at 37 °C in the dark. Images were captured using a Zeiss AX10 microscope. For quantitative analysis, HepG2/ADM cells were seeded in black 96-well microplates (2 × 10^4^ cells/well) and treated with MitoSOX Red. Then, a microplate reader (Beckman Coulter, USA) was used to measure the fluorescence intensity (Ex = 510 nm and Em = 580 nm for MitoSOX Red).

### Western blotting

Cells were collected by trypsinization and lysed in RIPA lysis buffer (containing 1 mM PMSF, 1 × phosphatase inhibitor, and 1 × protease inhibitor) to obtain total cellular protein. Cytosolic and mitochondrial protein extraction was performed using a digitonin-based method. HepG2/ADM cells were resuspended in cytosolic extraction buffer (250 mM sucrose, 70 mM KCl, 137 mM NaCl, 4.3 mM Na_2_HPO_4_, 1.4 mM KH_2_PO_4_, 100 μM PMSF, 10 μg/ml leupeptin, 2 μg/ml aprotinin, pH = 7.2) containing 200 μg/ml digitonin for 20 min on ice. After centrifugation, the supernatant was collected as the cytosolic protein fraction. Then, the cell pellets were lysed with RIPA lysis buffer (containing 1 mM PMSF, 1 × phosphatase inhibitor, and 1 × protease inhibitor) to obtain mitochondrial protein. A BCA assay was performed to quantify the protein concentrations. Cellular protein (30 µg) was separated on SDS-PAGE gels and then transferred onto polyvinylidene fluoride (PVDF) membranes. Incubation with primary antibodies was performed overnight at 4 °C. After that, the membranes were incubated with a secondary antibody for 1 h at room temperature. Immunoreactive proteins were visualized with an ECL chemiluminescence detection kit.

### Coimmunoprecipitation assay

HepG2/ADM cells were lysed with CO-IP lysis buffer (150 mM Tris, pH 7.6, 50 mM NaCl, 10 mM sodium pyrophosphate, 0.5% NP-40, 2 mM sodium orthovanadate, and 100 mM sodium fluoride) containing protease inhibitors on ice for 30 min. Cell debris was precipitated at 14,000 g and 4 °C for 10 min. A total of 500 μg of total protein was incubated with the indicated antibodies for 2 h at 4 °C. The immunoprecipitates were incubated with protein G-Sepharose (Thermo fisher) overnight at 4 °C. Then, the protein complexes were washed three times with CO-IP lysis buffer, eluted with 2 × Laemmli sample buffer at 95 °C and analyzed by western blotting. For ubiquitination detection under denaturing conditions, cells were lysed in buffer containing 10 mM Tris, pH 7.4, 1% SDS, 5 mM EDTA and 10 mM DTT with protease/phosphatase inhibitor cocktails and incubated for 30 min on ice. After lysis, the protein samples were diluted to 1 to 2 mg/ml with buffer containing 10 mM Tris, pH 7.4, 150 mM NaCl, 1% Triton X-100, 1 mM EDTA, 1 mM EGTA, and protease/phosphatase inhibitor cocktails. Immunoprecipitation was performed with the indicated antibody at a 1:50 dilution overnight at 4 °C. Then, the samples were incubated with protein G-Sepharose for 2 h at 4 °C. The immunoprecipitates were washed and eluted by incubation with 2 × Laemmli sample buffer at 100 °C and analyzed by western blotting.

### Tumor xenografts in nude mice

Animal studies were approved by the Laboratory Animal Ethics Committee of Jinan University (Guangzhou, China) (approval number: 2018416-209). HepG2/ADM cells (1 × 10^7^) mixed with Matrigel at a 2:1 volume ratio were injected subcutaneously into 6-week-old BALB/c nude mice (Vital River Laboratory Animal Technology, Beijing, China). When the tumor volumes reached approximately 300 mm^3^, the mice were divided randomly into two groups (*n* = 6 per group): vehicle and B5G1 (12.5 mg/kg). The mice were then administered vehicle intragastrically (5% DMSO and 10% HS 15 in saline) or B5G1 in saline containing 5% DMSO and 10% HS 15 once per day for the indicated number of days until the tumor volume of the vehicle group reached ~2000 mm^3^. Body weights and tumor volumes were measured every other day, and tumor volumes were calculated as (*a* × *b*^2^)/2, where *a* and *b* were the longest and shortest diameters of the tumors, respectively. At the end of the experiments, the mice were anesthetized by intraperitoneal (i.p.) injection of 5 ml/kg 1% pentobarbital sodium salt, and the tumors were removed, weighed, and photographed. The tumors were fixed in 4% paraformaldehyde until pathological examination.

### Histology and immunohistochemistry

Tumor tissues from the vehicle and B5G1 groups were embedded in paraffin and cut into 5 µm-thick sections. Then, the sections were stained with H&E. For immunohistochemical staining, the sections were incubated with anti-ki67, anti-cleaved caspase-3, anti-PINK1, anti-p-Parkin (Ser65), and anti-COX IV antibodies overnight at 4 °C and then with HRP-conjugated secondary antibodies. The sections were visualized using a DAB kit, and the images were observed using an Olympus BX 53 microscope.

### Statistical analysis

Each experiment was performed at least three times, and the data are shown as the mean ± standard deviation. Significant differences between two groups were determined using the two-tailed unpaired *t* test, and significant differences between more than two groups were evaluated using one-way ANOVA followed by Tukey’s post hoc test. Differences were considered significant when *P* < 0.05. All statistical data were calculated using GraphPad Prism software version 6.00 (GraphPad Prism Software, San Diego, CA, USA).

## Supplementary information

Supplementary S1

Supplementary S2

Supplementary S3

Supplementary Figure legends
