# Supplementary figures and images for: Inhibition of PINK1/Parkin-dependent mitophagy sensitizes multidrug-resistant cancer cells to B5G1, a new betulinic acid analog
Source: Cell Death Dis. 2019 Mar 8;10(3):232. doi: 10.1038/s41419-019-1470-z (PMC6408511; doi:10.1038/s41419-019-1470-z)

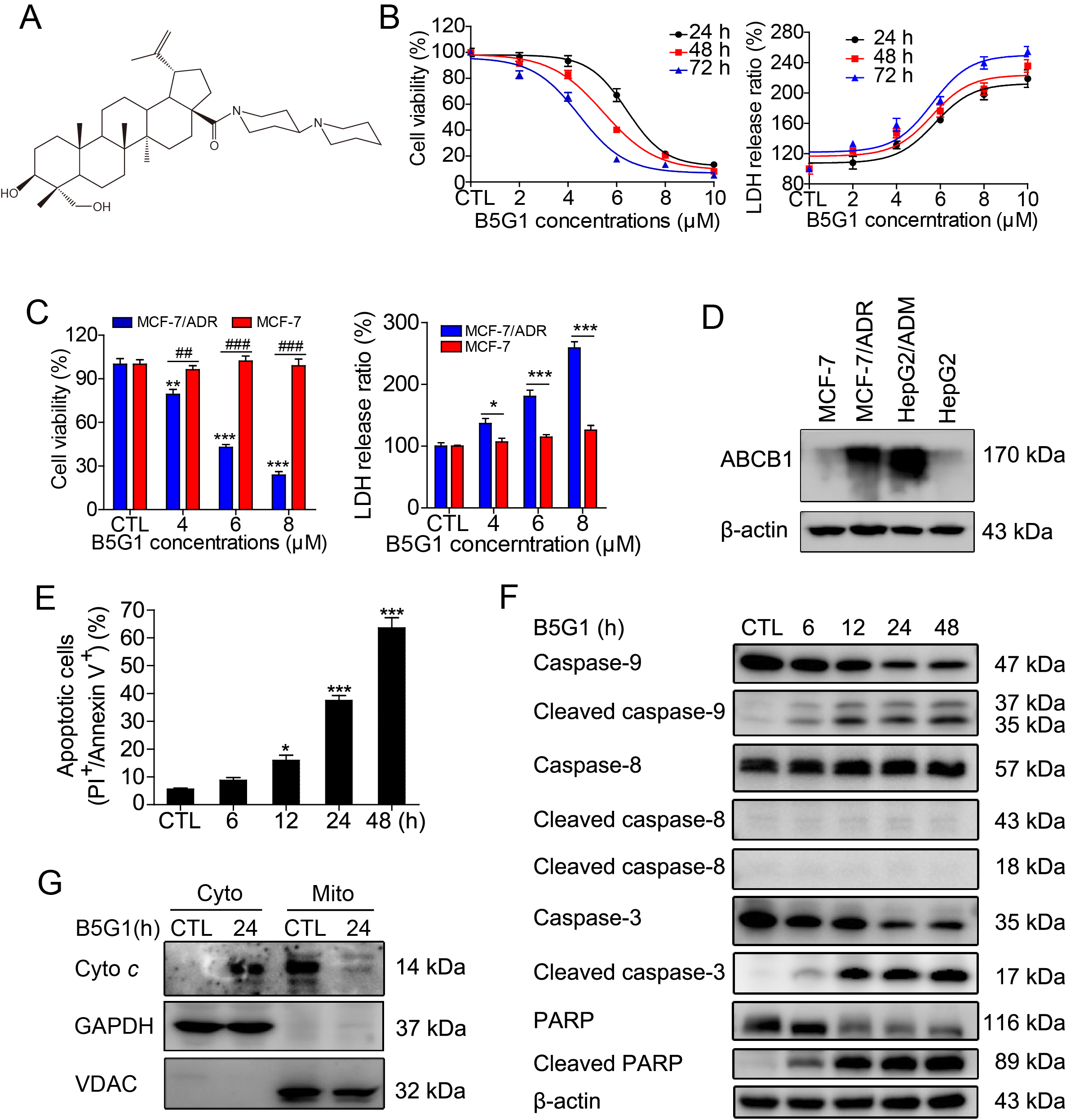

Supplement: Supplementary file 1 — Supplementary S1 [file 41419_2019_1470_MOESM1_ESM.jpg]

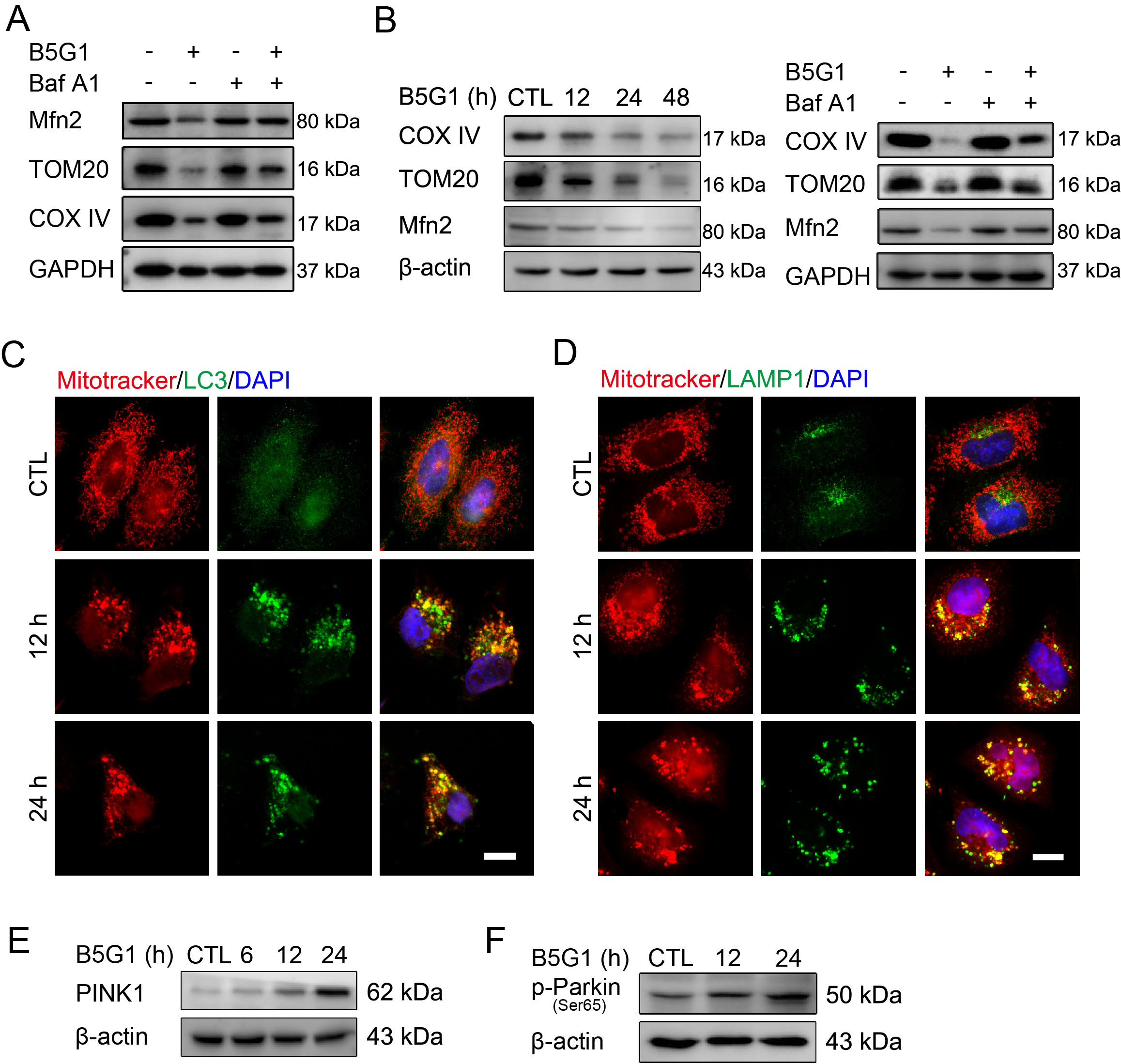

Supplement: Supplementary file 2 — Supplementary S2 [file 41419_2019_1470_MOESM2_ESM.jpg]

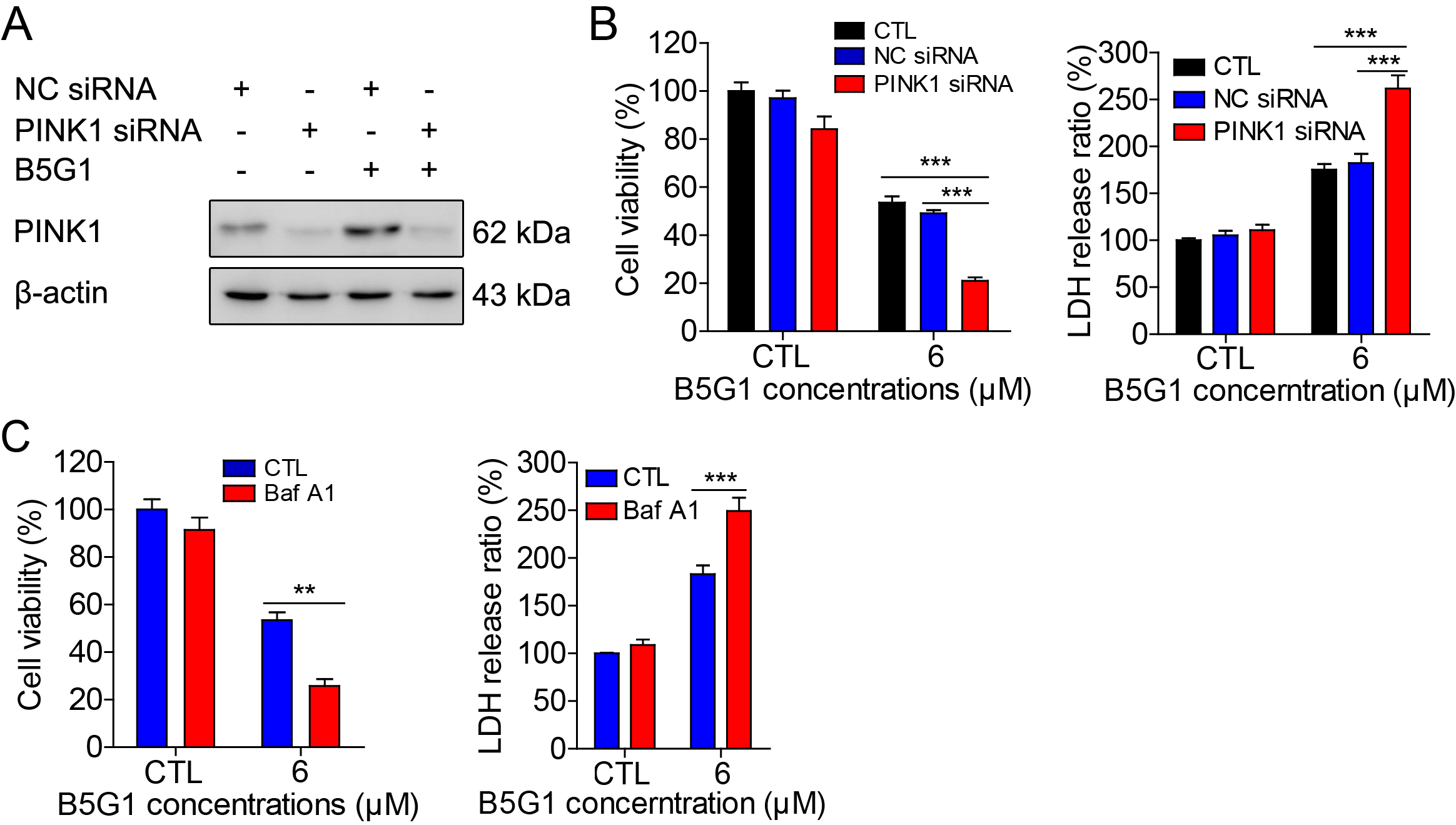

Supplement: Supplementary file 3 — Supplementary S3 [file 41419_2019_1470_MOESM3_ESM.jpg]
