# Supplementary material for: Inhibition of PINK1/Parkin-dependent mitophagy sensitizes multidrug-resistant cancer cells to B5G1, a new betulinic acid analog
Source: Cell Death Dis. 2019 Mar 8;10(3):232. doi: 10.1038/s41419-019-1470-z (PMC6408511; doi:10.1038/s41419-019-1470-z)
Supplement: Supplementary file 4 — Supplementary Figure legends [file 41419_2019_1470_MOESM4_ESM.docx]

**Supplementary figure legend:**

**Supplementary Figure S1.** B5G1 inhibits the proliferation of MCF-7/ADR cells *via* induction of mitochondrial apoptosis. (A) The chemical structure of B5G1. (B) MCF-7/ADR cells were treated with different concentrations of B5G1 for 24, 48 and 72 h. Cell viability was determined by MTT and LDH assay. (C) MCF-7/ADR and MCF-7 cells were treated with the indicated concentrations of B5G1 for 48 h, and cell viability was determined by MTT and LDH assay (n=3). ^**^*P* < 0.01, ^***^*P* < 0.001 *vs* CTL (MCF-7/ADR), ^##^*P* < 0.01, ^###^*P* < 0.001 (MTT assay); ^*^*P* < 0.05, ^**^*P* < 0.01, ^***^*P* < 0.001 (LDH assay). (D) The expression level of ABCB1 of MCF-7, MCF-7/ADR, HepG2/ADM and HepG2 cells were detected by Western blotting. β-actin was used as a loading control. (E) The apoptosis rates of MCF-7/ADR cells treated with B5G1 (6 μM) for indicated times were detected by flow cytometry (n=3). ^*^*P* < 0.05, ^***^*P* < 0.001 *vs* CTL. (F) The expression level of apoptosis-related proteins of MCF-7/ADR cells treated with B5G1 (6 μM) for the indicated times were analyzed by Western blotting. β-actin was used as a loading control. (G) Cell lysates of MCF-7/ADR cells treated with B5G1 (6 μM) for 24 h were divided into cytoplasmic fractions and mitochondrial fractions. Cyto *c* translocation was measured by Western blotting. GAPDH and VDAC were used as loading controls for cytoplasm and mitochondria, respectively.

**Supplementary Figure S2.** B5G1 induces mitophagy in MCF-7/ADR cells. (A) HepG2/ADM cells were exposed to B5G1 (6 μM) in the presence or absence of Baf A (200 nM) for 24 h. Mitochondrial proteins expression levels were measured by Western blotting. GAPDH was used as a loading control. (B) MCF-7/ADR cells were treated with B5G1 (6 μM) for the indicated times, expression level of mitochondrial proteins were analyzed by Western blotting. β-actin was used as a loading control (left panel). MCF-7/ADR cells were treated with B5G1 (6 μM) in the presence or absence of Baf A (200 nM) for 24 h. Mitochondrial proteins expression levels were measured by Western blotting. GAPDH was used as a loading control (right panel). (C-D)After treatment with B5G1 (6 μM) for the indicated times, MCF-7/ADR cells were stained with MitoTracker red (200 nM) and immunostained with an LC3 or LAMP1 antibody. Mitochondrial colocalization with LC3 or LAMP1 was observed by a fluorescence microscope. Magnification: 630×; scale bar: 10 μm. (E-F) MCF-7/ADR cells were treated with B5G1 (6 μM) for the indicated times, expression level of PINK1 and p-Parkin (Ser65) were analyzed by Western blotting. β-actin was used as a loading control.

**Supplementary Figure S3.** Inhibition of mitophagy sensitizes MCF-7/ADR cells to B5G1 treatment. (A) MCF-7/ADR cells were pretreated with NC or PINK1 siRNA for 24 h, followed by treatment with B5G1 (6 µM) for another 12 h. PINK1 expression levels were detected by Western blotting. β-actin was used as a loading control. (B) MCF-7/ADR cells were transfected with NC or PINK1 siRNA and then treated with B5G1 (6 µM) for 48 h. Cell viability was measured by MTT and LDH assay (n=3). ^***^*P* < 0.001. (C) MCF-7/ADR cells were pretreated with Baf A1 (200 nM) for 1 h, followed by treatment with B5G1 (6 µM) for 48 h. Cell viability was measured by MTT and LDH assay (n=3). ^**^*P* < 0.01, ^***^*P* < 0.001.
